# A case of Henoch-Schönlein purpura associated with posterior reversible encephalopathy and review of litereature

**DOI:** 10.1186/1546-0096-12-S1-P358

**Published:** 2014-09-17

**Authors:** Kyu Yeun Kim, Mo Kyung Jung, Ki Hwan Kim, Dong Soo Kim

**Affiliations:** 1 Pediatrics, Division of Rheumoatology, College of Medicine, Yonsei University, Severance Children’s Hospital, Seoul, Korea, Republic Of; 2 Pediatrics, Division of Rheumatology, College of Medicine, Yonsei University, Severance Children’s Hospital, Seoul, Korea, Republic Of

## Introduction

Henoch-Schönlein purpura (HSP) is a vasculitis that involves small vessels and seen in children predominantly. Main symptoms are purpuric rashes on body, especially on lower extremities, arthralgia, abdominal pain, and nephritis. Uncommonly, nervous system can be involved.

## Objectives

PRES can occur in HSP infrequently. Here we will review our case, a first reported Korean case, and compare with published cases to find out which symptoms and signs should be aware.

## Methods

First, our case is reviewed retrospectively by electrical medical record. And then, we searched Pubmed database, terms including HSP, PRES, RPLS, and encephalopathy. We compared collected cases and our case.

## Results

A 8-year-old girl visited our hospital complaining abdominal pain and purpuric rash on lower extremities and buttock. On hospital day 7, there were two brief events of generalized tonic-clonic seizure. She complained dizziness and blurred vision. Brain MRI demonstrated increased signal intensity in the cortex and subcortical white matter, in the parietooccipital area, and impression was PRES. She was discharged on day 19 without any complication. Table 1.

**Table 1:** 

	Age	Sex	Main symptom	Neurologic symptom	Blood pressure	Thrapy
Our case	8yr	F	Abdominal pain, Blood tinged stool, Purpura	GTC, Blurred vision	144/86	MPT pulse Tx, anti-hypertensive drug, anti-convulsive drug

Dasarathi, et al.	11yr	F	Abdominal pain, vomiting, purpura	GTC, complete loss of vision	112/67	supportive Tx

Sivrioglu, et al.	5yr	F	purpura, arthralgia, abdominal pain	headache seizure	180/110	anti-hypertensive drug, anti-convulsive drug, dialysis

Sasayama, et al.	13yr	F	abdominal pain, purpura	generalized seizure, cortical blindness	180/120	MTP pulse Tx, anti-hypertensive drug

Fuchigami, et al.	7yr	F	abdominal pain, arthralgia, purpura	sudden loss of vision GTC	190/100	PL, anti-hypertensive drug, anti-convulsive drug

Woolfenden et al.	10yr	M	fever, RLQ pain, bloody diarrhea	Bi-temporal headache, nausea, vomiting bilateral visual loss seizure	not known	MTP pulse Tx, anti-convulsion drug

Ozcakar et al.	10yr	M	fever, palpable purpra, arthralgia	seizure	130/90	steroid, anti-convulsive Tx, anti-hypertension drug

Endo et al.	7yr	F	abdominal pain, purpura	seizure unconsciousness loss of vision	190/100	PL, anti-hypertensive drug, anti-convulsive drug

## Conclusion

In HSP patients, hemodynamic change due to severe hypertension and renal insufficiency, and CNS vasculitis can cause PRES. JI Shin suggests IL-6 and VEGF can play a role in this situation. Neurological involvement is not common in HSP. But when patients complain headache or blurred vision, it can be manifestation of CNS lesion, including PRES. Adequate assessment and manage should be performed to avoid neurologic sequela.

## Disclosure of interest

None declared

